# Twelve-oxoeicosatetraenoic acid-induced fetal membrane release improves postpartum ovarian function, milk production, and blood plasma biochemical parameters in cows

**DOI:** 10.5713/ab.22.0443

**Published:** 2023-02-26

**Authors:** Hachiro Kamada, Yoshitaka Matsui

**Affiliations:** 1 Institute of livestock and Grassland Science, NARO, Tsukuba, 305-0901, Japan; 2 Present address: Tohoku Agricultural Research Center, NARO, Shimokuriyagawa, Morioka, 020-0198, Japan; 3 Hokkaido Research Organization, Agricultural Research Department, Dairy Research Center, Nakashibetsu, Hokkaido, 086-1135, Japan

**Keywords:** Cow, 12-Oxoeicosatetraenoic Acid, Placenta, Progesterone

## Abstract

**Objective:**

We aimed to determine the effects of 12-oxoeicosatetraenoic acid (12-KETE)-induced placenta release on the performance of mother cows (milk yield, ovarian function, and blood plasma biochemical properties).

**Methods:**

Experimental treatments were as follows: i) natural delivery including natural placental release (control cows); ii) induced calf delivery with placental retention (RP cows); and iii) induced calf delivery and 12-KETE-induced placental release (KE cows). Delivery in pregnant KE cows was induced with dexamethasone and prostaglandin. These cows were injected with 12-KETE after calf discharge, resulting in the release of the fetal placenta. RP cows were not treated with 12-KETE after inducing delivery, resulting in placental retention.

**Results:**

The milk yield in RP cows during the first 50 days after delivery was significantly lower than that in control cows (p<0.05), whereas KE cows exhibited a similar milk yield to that of control cows. The postpartum plasma progesterone levels of control cows increased 14 days after delivery on average; however, its increase was delayed by 10 days in RP cows. Meanwhile, the 12-KETE treatment (KE cows) brought the timing of progesterone increase forward to the normal level (control cows). Among the 20 biochemical parameters examined, the total cholesterol levels in blood plasma 14 days after delivery were lower in RP cows than that in the other two treatment groups (control cows and KE cows) (p<0.05). In addition, the plasma level of haptoglobin tended to be low in cows that discharged their placentas shortly after delivery.

**Conclusion:**

These findings indicate that 12-KETE treatment can alleviate the disorder caused by placental retention.

## INTRODUCTION

Peripartum accidents can adversely affect the reproductive performance of cows [1]. For example, such problems cause an increased mortality rate among newborn calves [2] and a decreased conception rate among cows [3,4]. Although the care of deliveries of cows is important to decrease peripartum accidents, delivery care at midnight is especially strenuous for livestock farmers, and its execution rate is generally low [5]. Thus, livestock farmers require techniques that can safely induce daytime delivery in cows. In addition, the ideal delivery induction techniques should not result in retained placenta (RP) in cows.

Nighttime feeding has been proposed as a method for inducing daytime delivery [6–8]; however, it is not possible to predict a real delivery date after treatment. Furthermore, this technique cannot be used for cows suffering from a prolonged gestation, whose delivery is delayed by more than one week from the expected date of delivery, as it cannot induce a delivery to prevent the overgrowth of the fetus, which can result in hard labor. On the other hand, the administration of glucocorticoids or prostaglandins (PGs) to pregnant cows can induce fetal delivery. By regulating the timing and dose of hormone injection, we can induce daytime delivery among cows [9]. This method induces delivery within about 1.5 to 2 days after the administration of hormone [10]; however, the incidence of RP is high [11,12]. It is known that fetal discharge, which is the first stage of the delivery, is triggered by glucocorticoids or PGs [13]; however, a signal for the post-delivery release of the unneeded placenta has not yet been identified. In a previous study, we identified 12-oxoeicosatetraenoic acid (12-KETE) as a potential signal for placental release after calf discharge [14,15]. Moreover, we succeeded in artificially inducing calf discharge, which was followed by the release of the placenta, via the administration of 12-KETE to pregnant cows. In this study, the effects of 12-KETE-induced placental release on the postpartum performance of mother cows were investigated to show the availability of placenta discharge induced by 12-KETE. The metabolic rate of the injected 12-KETE was also measured. This is an important piece of data in the development of new drugs to induce placenta release.

## MATERIALS AND METHODS

### Animal treatment

Pregnant Holstein cows (n = 26) were subcutaneously administered 2.5 or 5 mg dexamethasone (Kyoritsu Seiyaku, Tokyo, Japan) at 7:00 in the morning, 8 days before their expected delivery date. In addition, after 24 h (at 7:00 in the morning of the next day) 50 mg of PG F2alpha (dinoprost, Zoetis, Tokyo, Japan) was administered to these cows intramuscularly to induce fetal delivery. When the delivery induction time (the period from the injection of PG to fetal discharge) was more than 30 h, a 2.5, 5, or 7 mg of 12-KETE was administered intrajugularly to the mother cows at 2 or 4 h after fetal discharge (KE cows). Under the abovementioned conditions, the cows that were not treated with 12-KETE suffered from RP (RP cows). The cows that did not discharge their placenta within 12 h after calf discharge were regarded as RP cows. The control cows (Holstein, n = 19) were not injected with hormones or 12-KETE, and underwent natural delivery. The term “Natural-RP” (Holstein, n = 8) shown in Figure 3A means that the cows suffered from RP after natural delivery. All cows were kept in our institute and provided with an adequate amount of the same feed (total mixed ration and Italian ryegrass hay) according to the Japanese Feeding Standard for Dairy Cattle [16]. The comparisons of milk production, blood plasma biochemical parameters, and high performance liquid chromatography (HPLC) analyses were the results of primiparous cows.

Blood samples were collected from each cow intrajugularly using a heparinized tube in the morning for every two days, between 0–50 days after delivery. After centrifugation, the blood plasma was stored at −40°C until further analyses were conducted. The milk yields of the primiparous cows were measured twice daily (control cows, n = 15; RP cows, n = 7; KE cows, n = 5).

All procedures were approved by the Animal Care and Use Committee of the Institute of Livestock and Grassland Science, NARO.

### Determination of plasma progesterone levels

The postpartum progesterone (P4) levels in the blood plasma of Holstein cows were measured using the Access 2 Immunoassay System (Beckman Coulter, Brea, CA, USA). The intra-assay and inter-assay coefficients of variation were 4.84% and 9.35%, respectively (control cows, n = 11; RP cows, n = 15; KE cows, n = 5; natural RP cows, n = 8).

### Blood biochemical parameters

The biochemical parameters (the plasma levels of glutamic oxaloacetic transaminase [GOT], glutamic pyruvic transaminase [GPT], lactate dehydrogenase [LDH], alkaline phosphatase [ALP], total protein [TP], albumin [Alb], blood urea nitrogen [BUN], Glu, total cholesterol [T-cho], non-esterified fatty acid [NEFA], triglyceride [TG], inorganic phosphorus [iP], total ketone body, beta-hydroxybutyric acid [BHB], Fe, Ca, Na, Cl, Mg, and K) of primiparous Holstein cows were determined at the Iwamizawa Medical Laboratory (Hokkaido, Japan). Thereafter, T-cho levels of these cows were again measured using a commercial kit (Cholesterol E test, Wako, Osaka, Japan) (control cows, n = 9; RP cows, n = 6; KE cows, n = 5).

### Determination of plasma haptoglobin levels

The amount of haptoglobin (an early indicator of metritis [17]) in the postpartum blood plasma of Holstein cows was measured using a bovine haptoglobin ELISA test kit (Life Diagnostics, Inc., West Chester, PA, USA). Plasma samples were diluted by 4,000- or 8,000-fold. A 100 μL portion of the standards or a diluted sample was dispensed into microtiter wells and incubated on a microplate shaker at room temperature for 45 min. After incubation, the wells were washed and emptied five times with a wash solution. Furthermore, 100 μL of enzyme-conjugated reagent was added into each well and incubated on a microplate shaker at room temperature for 45 min. These wells were again washed and emptied five times, and 100 μL of TMB (3, 3′, 5, 5′-tetramethylbenzidine) Reagent was added. After gentle mixing at room temperature for 20 min, the enzyme reaction was stopped by adding 100 μL of stop solution. Thereafter, the optical density of each well was measured at 450 nm using a micro-plate reader (BIO-RAD, Hercules, CA, USA) (control cows, n = 10; RP cows, n = 10; KE cows, n = 5).

### Determination of plasma 12-KETE levels

The injected 12-KETE was determined to compare 12-KETE peaks in the blood plasma of cows that underwent induced and natural deliveries. After injecting 5 mg of 12-KETE into the jugular vein after inducted delivery using PG, blood samples of five primiparous Holstein cows were collected using a blood catheter in the opposite vein at 0, 30, 60, 90, 120, 150, 180, 240, and 300 s after injection. The blood plasma was stored at −80°C until further analysis. The determination of 12-KETE was conducted using a previously described method (HPLC analyses) [14].

### Statistical analysis

Milk, P4, and T-cho data were analyzed via analysis of variance using the general linear models procedure (SAS, version 9.2; SAS Institute, Cary, NC, USA). Additionally, haptoglobin data were analyzed using the MIXED procedure in SAS. When significant treatment effects were found, Duncan’s multiple range test was used to determine the significance of any differences between the treatment groups. Results were considered significant at p<0.05.

## RESULTS

### Delivery induction

**Figure 1:**
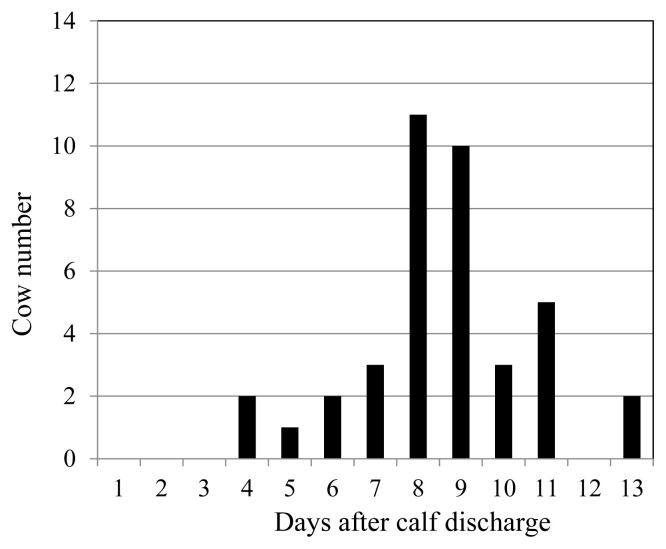
Days until placenta discharge in the cows suffering from retained placenta.

When PG was injected into pregnant cows 7 days before their expected delivery dates, approximately 90% of the multiparous cows (18/21) and 70% of the primiparous cows (22/32) suffered from RP. However, 80% of RP cows discharged their denatured placenta 8 days or later after the delivery (Figure 1). Meanwhile, cows injected with an adequate amount of 12-KETE discharged their placenta 4.7±1.9 h after treatment on average. The mortality rate of newborn Holstein calves was 1.7%. Furthermore, 81% of the deliveries occurred during normal working hours for farmers (6:00 to 20:00).

### Milk yield

**Figure 2:**
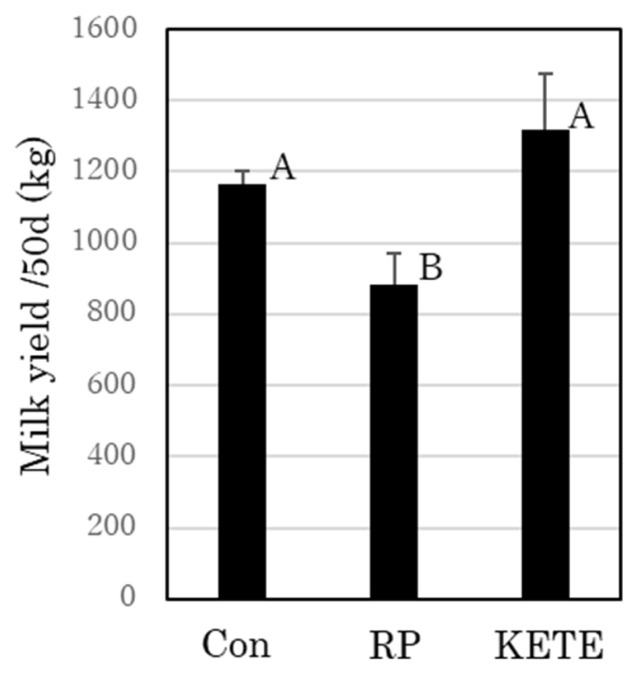
Recovery of milk yield after 12-oxoeicosatetraenoic acid (12-KETE)-induced fetal membrane release. Con (n = 15), RP (n = 7), KETE (n = 5). Data is presented as mean±standard error (SE). ^A,B^ Different capital letters indicate a statistically significant difference among the treatments (p<0.05). Con, cows that delivered naturally and without suffering from RP; RP, cows that underwent induced delivery and suffered from RP; KETE, cows injected with 12-KETE and did not suffer from RP.

The mean milk yield of the control cows during the first 50 days after delivery was 1,165.5±35.1 kg. RP cows exhibited decreased milk production from about 9 days after delivery. Thereafter, their milk production gradually recovered; however, their mean milk yield was lower (884.8±83.6 kg) than that of control cows (p<0.05). In contrast, KE cows did not show any reduction in milk production compared to that in the control cows (1,315.7±160.8 kg; Figure 2).

### Postpartum plasma progesterone concentration

**Figure 3:**
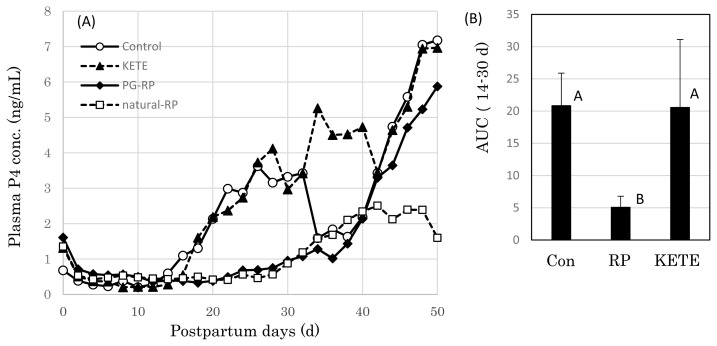
Effects of induced fetal membrane release on the postpartum plasma progesterone (P4) concentration. (A) P4 profile, (B) the area under the curve (AUC) of P4 during 14–30 day after delivery. Con (n = 11), PG-RP (n = 15), KETE (n = 5), natural-RP (n = 8). Data is presented as mean±standard error (SE). ^A,B^ Different capital letters indicate a statistically significant difference among the treatments (p<0.05). Con, cows that delivered naturally and without suffering from RP; PG-RP, cows that underwent induced delivery and suffered from RP; KETE, cows injected with 12-KETE and did not suffer from RP; natural-RP, cows with naturally occurring RP.

The mean concentration of plasma progesterone (P4) of the control cows increased gradually from postpartum Day 14 (Day 0 = date of delivery); however, in RP cows, this increase was delayed by 10 days (Figure 3A). Meanwhile, in KE cows the plasma P4 level started to increase at the same time as that in the control cows. The area under the curve (AUC) of the P4 concentration between Day 14 and 30 was significantly lower in RP cows than those in the other two treatments (control and KE cows) (p<0.05) (Figure 3B). KE cows showed an early increase in blood P4 concentration when compared with that of RP cows.

### Biochemical parameters of blood plasma

**Figure 4:**
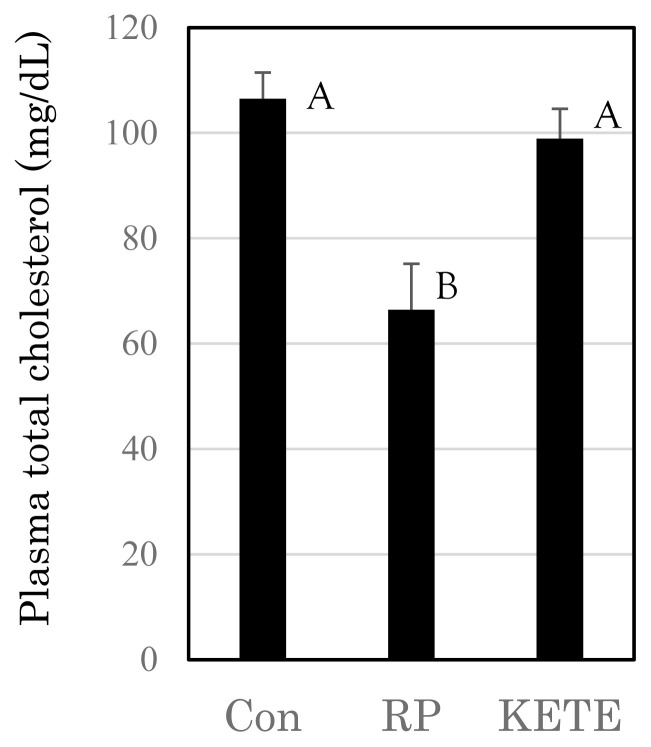
Effects of induced fetal membrane release on the plasma total cholesterol levels 14 days after delivery. Con (n = 9), RP (n = 6), KETE (n = 5). Data is presented as mean±standard error. ^A,B^ Different capital letters indicate a statistically significant difference among the treatments (p<0.05). Con, cows that delivered naturally and without suffering from RP; RP, cows that underwent induced delivery and suffered from RP; KETE, cows injected with 12-KETE and did not suffer from RP.

Although the plasma levels of GOT, GPT, LDH, ALP, TP, Alb, BUN, Glu, TG, NEFA, iP, total ketone body, BHB, Fe, Ca, Na, Cl, Mg, and K did not differ among the treatment groups, on Day 14 the plasma levels of total cholesterol were significantly lower in RP cows than those in the other treatments (p<0.05) (Figure 4).

### Plasma haptoglobin level

**Figure 5:**
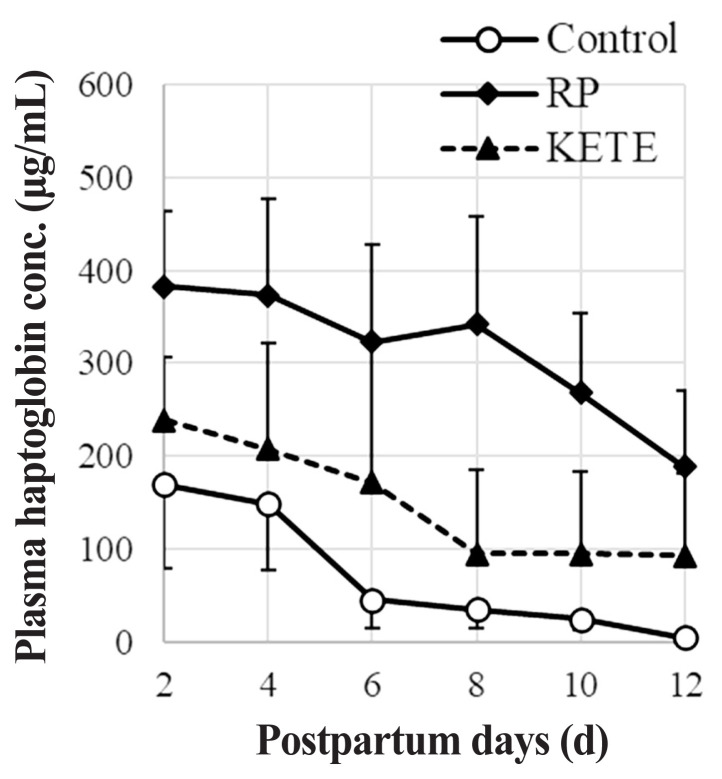
Effect of induced fetal membrane release on the postpartum plasma haptoglobin concentration. Con (n = 10), RP (n = 10), KETE (n = 5). Data is presented as mean±standard error. Con, cows that delivered naturally and without suffering from RP; RP, cows that underwent induced delivery and suffered from RP; KETE, cows injected with 12-KETE and did not suffer from RP.

The postpartum plasma haptoglobin levels of cows were not necessarily related to the retention of the placenta in their uterus; however, the cows whose placenta were discharged shortly after delivery tended to exhibit early reductions in the concentration of haptoglobin (Figure 5). On the other hand, cows that could not quickly discharge their placenta (RP cow) maintained high blood haptoglobin level. In the control, RP, and KE cows, 80%, 40%, and 80% of the cows, respectively, had plasma haptoglobin concentrations of <10 μg/mL on Day 12 after delivery.

### Metabolism of the injected 12-oxoeicosatetraenoic acid

**Figure 6:**
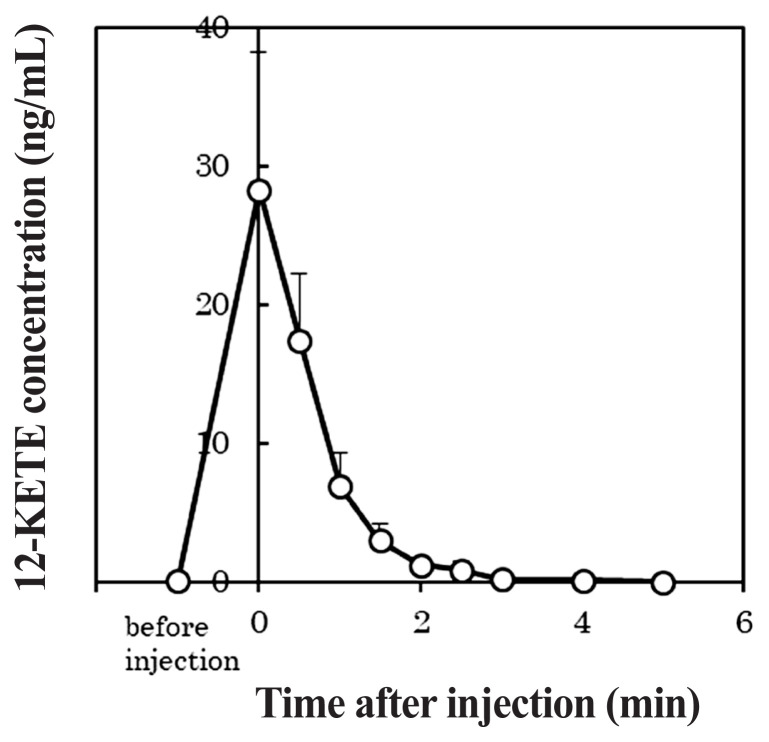
Metabolism of injected 12-oxoeicosatetraenoic acid (n = 5). Data is presented as mean±standard error.

Figure 6 shows the profile of the plasma 12-KETE concentration when 5 mg of 12-KETE was injected into primiparous cows (n = 5). The average concentration of 12-KETE in these cows had reached its maximum immediately after the injection and then rapidly decreased. Furthermore, the 12-KETE disappeared from the blood plasma within 3 min post injection. The maximum 12-KETE concentration in plasma was 28.29 ng/mL on average.

## DISCUSSION

The induction of delivery using PG or glucocorticoid is of limited use for livestock farmers because of the high risk of RP. Because this method contains only a signal for fetal discharge and has no signal for placental discharge, the placenta does not discharge from uterus. While a signal for placenta discharge was unknown, there was information about a mediator candidate concerning fetal placenta separation from maternal tissue. The contribution of matrix metalloproteinase (MMP: an *in vivo* collagen-degrading enzyme) on placenta discharge was suggested [18]. In the placenta of RP cows, specific MMPs were lower than those of non-RP cows. We also reported that the MMP of bovine trophoblasts was activated by 12-KETE [15]. Actually, Eiler et al [19] succeeded in placenta discharge using the injection of collagenase solution via umbilical arteries, however; this technique seemed to need high skill levels. In a previous study, we found evidence that 12-KETE acted as a signal for placental discharge in cows, and succeeded in the induction of placental discharge in >80% of cases via a 12-KETE intravenous injection (a simple method) in delivery-induced dams [14]. This report showed that the induction of placenta release using 12-KETE improved the postpartum performance of dams in which artificial delivery was induced.

Cows suffering from RP frequently develop fevers and decreased feed intake, which leads to lower milk production and, in the worst case, abomasal displacement [20–22]. Thus, livestock farmers prefer to take out the placenta from the uterus of the mother cows as soon as possible. However, the manual removal of a retained placenta is not recommended [23] because of the risks of pathogenic infection or injury of the uterus if the attachment between the cotyledon and caruncle is hard. A 12-KETE-induced placenta release can solve such problems. In a previous study, 12-KETE was detected in the blood plasma of postpartum cows prior to a natural discharge of the placenta [14], thus the induction of placenta release with the injection of 12-KETE was considered to reappear the physiological phenomenon. The present study demonstrated that inducing placental release by injecting cows with 12-KETE improved the milk production that reduced during the early lactation period, which is a drawback of the conventional delivery induction method using hormones.

When RP in a postpartum cow was left untouched, it took 4 to 13 days for the denatured placenta to discharge from the uterus naturally. Based on this observation, a delay in the recovery of the uterus can be expected in cows with RP. Buso et al [24] reported a negative effect of RP on reproductive efficiency in crossbred dairy cows. RP increased the calving to conception interval and number of artificial insemination per conception. Holt et al [25] showed the delayed increases in postpartum plasma progesterone (P4) concentrations in RP cows, which was consistent with our results. The current study showed that the induction of placental release using 12-KETE recovered the postpartum corpus luteum (CL) function to the same level as that observed in the control cows, as the AUC of the mean P4 concentrations is considered to be an indicator of the presence/absence of functional CL. Darwash et al [26] reported that the early recovery of ovarian function increased the subsequent fertility of cows. The early recovery of luteal function observed in this study could contribute to the fertility of cows. Although the influence of 12-KETE-induced placental release on the reproductive parameters (e.g., on the conception rate and conception day) is an interesting subject, we do not have sufficient data about these issues at present. However, in this study, all cows whose placental release was induced using 12-KETE subsequently became pregnant and delivered calves; hence, it is likely that the 12-KETE treatment does not have any adverse effects on the reproductive performance of cows.

Skinner et al [27] and Pohl et al [28] reported that the blood haptoglobin levels of cows with RP showed increases. The current study also showed that most of the cows with RP had high haptoglobin levels. The increase in haptoglobin level may not be entirely caused by RP; however, the cows in the control and 12-KETE treatment groups, who discharged their placentas shortly after delivery, tended to show low plasma haptoglobin levels. In addition, dairy cows that were diagnosed with clinical metritis had higher circulating concentrations of haptoglobin [29]; hence, the delayed recovery of CL function seen in the RP cows might have been caused by the continuation of metritis due to the RP. It is possible that the induction of placental release using the 12-KETE treatment suppressed metritis, and thus, most of the cows treated with 12-KETE could recover their post-delivery CL function sooner.

Trevisi et al [30] reported that cows with RP displayed lower blood cholesterol levels than those of control cows, which was consistent with our data. Because P4 is synthesized from cholesterol, a shortage of cholesterol might have a negative effect on P4 synthesis. Moreover, low blood cholesterol levels may be caused by decreased dietary intake.

In the current study, the maximum concentration of 12-KETE in blood plasma was approximately 1.7 times the peak value seen after natural delivery (16.8 ng/mL [14]). Although the 12-KETE peak in plasma was maintained for several hours after natural delivery [14], the injected 12-KETE disappeared rapidly in the present study. It is probable that the catabolic rate of injected 12-KETE is so fast, meanwhile, *in vivo*, 12-KETE may be secreted continuously, causing its concentration in plasma to be maintained for long time.

The induction of placenta release via 12-KETE administration suppressed the inflammation of the uterus, resulting in early recovery of ovarian functions, and avoided a decrease in early milk production due to low feed intake. In future studies, we will not only apply this method (the induction of calf delivery and placental release) to the cases of prolonged gestation, but also use it to induce daytime delivery. Delivery induction methods using hormones (PG or glucocorticoid) can control the timing of calf discharge and create the possibility to perform a daytime delivery; however, they have the serious drawback of RP. This weakness could be overcome by inducing placental release with 12-KETE. One of the benefits of daytime delivery is that farmers can adequately manage both the dam (dealing with a hard labor) and calf (rapid colostrum feeding), which would help to reduce calf mortality. Actually, the mortality rate of newborn Holstein calves in this study was lower than the value (5% to 10%) described in previous reports [31,32], and the frequency of daytime delivery was high (81%).

This study showed that placenta release induced via 12-KETE treatment improved milk production, blood cholesterol, blood haptoglobin and CL function. This method will contribute to the management of pregnant cows.
